# Antioxidant Structure–Activity Relationship Analysis of Five Dihydrochalcones

**DOI:** 10.3390/molecules23051162

**Published:** 2018-05-12

**Authors:** Xican Li, Ban Chen, Hong Xie, Yuhua He, Dewei Zhong, Dongfeng Chen

**Affiliations:** 1 School of Chinese Herbal Medicine, Guangzhou University of Chinese Medicine, Waihuan East Road No. 232, Guangzhou Higher Education Mega Center, Guangzhou 510006, China; imchenban@foxmail.com (B.C.); xiehongxh1@163.com (H.X.); skywalkervpn@126.com (Y.H.); zhongdexinwei@163.com (D.Z.); 2 Innovative Research & Development Laboratory of TCM, Guangzhou University of Chinese Medicine, Waihuan East Road No. 232, Guangzhou Higher Education Mega Center, Guangzhou 510006, China; 3 School of Basic Medical Science, Guangzhou University of Chinese Medicine, Waihuan East Road No. 232, Guangzhou Higher Education Mega Center, Guangzhou 510006, China; 4 The Research Center of Basic Integrative Medicine, Guangzhou University of Chinese Medicine, Waihuan East Road No. 232, Guangzhou Higher Education Mega Center, Guangzhou 510006, China

**Keywords:** antioxidant, dihydrochalcone, naringin dihydrochalcone, neohesperidin dihydrochalcone, phloretin, phloridzin, trilobatin

## Abstract

The study determined the comparative antioxidant capacities of five similar dihydrochalcones: phloretin, phloridzin, trilobatin, neohesperidin dihydrochalcone, and naringin dihydrochalcone. In the ferric-reducing antioxidant power (FRAP) assay, the antioxidant activities of pairs of dihydrochalcones had the following relationship: phloretin > phloridzin, phloretin > trilobatin, trilobatin > phloridzin, trilobatin > naringin dihydrochalcone, and neohesperidin dihydrochalcone > naringin dihydrochalcone. Similar relative antioxidant levels were also obtained from 1,1-diphenyl-2-picryl-hydrazl radical (DPPH•)-scavenging, 2,2′-azino-bis(3-ethylbenzo-thiazoline-6-sulfonic acid) (ABTS•^+^)-scavenging, and superoxide radical (•O_2_^−^)-scavenging assays. Using ultra-performance liquid chromatography coupled with electrospray ionization quadrupole time-of-flight tandem mass spectrometry (UPLC−ESI−Q−TOF−MS/MS) analysis for the reaction products with DPPH•, phloretin, phloridzin, and trilobatin were found to yield both dihydrochalcone-DPPH adduct and dihydrochalcone-dihydrochalcone dimer, whereas naringin dihydrochalcone gave a naringin dihydrochalcone-DPPH adduct, and neohesperidin dihydrochalcone gave a dimer. In conclusion, the five dihydrochalcones may undergo redox-based reactions (especially electron transfer (ET) and hydrogen atom transfer (HAT)), as well as radical adduct formation, to exert their antioxidant action. Methoxylation at the *ortho*-OH enhances the ET and HAT potential possibly via p-π conjugation, whereas the glycosylation of the –OH group not only reduces the ET and HAT potential but also hinders the ability of radical adduct formation. The 2′,6′-di-OH moiety in dihydrochalcone possesses higher ET and HAT activities than the 2′,4′-di-OH moiety because of its resonance with the adjacent keto group.

## 1. Introduction

Dihydrochalcones are an unusual group of natural antioxidants. To date, fewer than 100 dihydrochalcones have been isolated from plants (especially Chinese herbal medicines) [1,2]. Nevertheless, the bioactivity of dihydrochalcones has attracted increasing interest from scientists in recent years. For example, phloretin, a typical dihydrochalcone, has been investigated for its anti-inflammatory and hepatoprotective effects in mice models [3,4], as well as neuroprotective effects in cellular models [5]. Its glucoside phloridzin (i.e., phloretin 2′-*β*-d-glucoside) has also been suggested to possess neuroprotective and cytoprotective effects [5,6]. Another glucoside of phloretin, trilobatin (i.e., phloretin 4′-*β*-d-glucoside), has also been shown to prevent inflammation by suppressing the NF-κB signaling pathway [7]. The three dihydrochalcones however were recently reported to coexist in apples, and to be responsible for the antidiabetic effect of apples [8].

**Figure 1:**
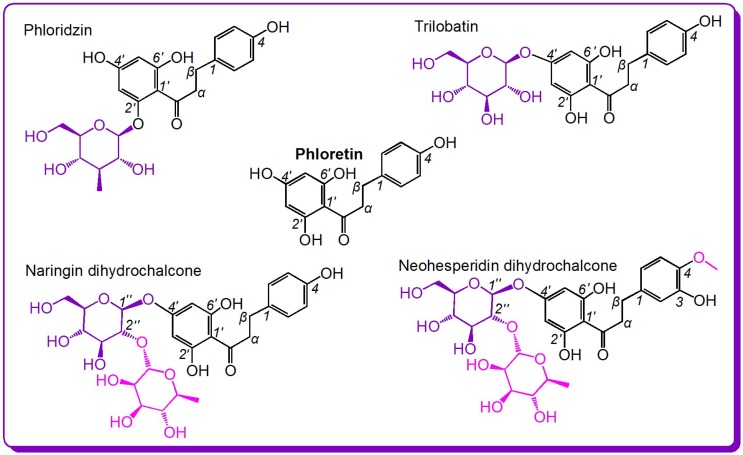
The structures of the five dihydrochalcones.

From the perspective of free radical biology and medicine, these bioactivities may be associated with the antioxidant capacities of these compounds [9,10]. In fact, phloretin, phloridzin, and neohesperidin dihydrochalcone have been found to show antioxidant effects [4,6,11,12,13]. Dihydrochalcones have even been proven to be stronger antioxidants than the corresponding flavones owing to the presence of the 2′-OH group [14]. Nevertheless, there have been no studies focusing on the antioxidant structure–activity relationship of the dihydrochalcone family. Thus, the study selected five dihydrochalcones for such investigation (Figure 1).

As shown in Figure 1, of the five dihydrochalcones, the simplest is phloretin; the other four are its derivatives, including phloridzin, trilobatin, neohesperidin dihydrochalcone, and naringin dihydrochalcone. Thus, their structures are similar, and they can act as ideal references for an antioxidant structure–activity relationship analysis of the dihydrochalcone family. For example, a comparison of phloretin and phloridzin (or of phloretin and trilobatin) can identify whether the glycosylation affects the antioxidant capacity, a comparison of phloridzin and trilobatin can identify the effect of the glycosylation site (or relative positions of di-OH), a comparison of trilobatin and naringin dihydrochalcone can identify the effect of the degree of glycosylation, and a comparison of naringin dihydrochalcone and neohesperidin dihydrochalcone can show the effect of the –OCH_3_ group on the antioxidant capacity.

It must be emphasized that the so-called antioxidant action is not a simple chemical reaction. Sometimes, multiple and complicated mechanisms are involved. Thus, to obtain a clear antioxidant structure–activity relationship, some well-known antioxidant assays were used for the study. For example, the ferric-reducing antioxidant power (FRAP) assay, a well-known electron-transfer (ET) reaction-based assay, was used in this study [15]. Thus, it can also provide an understanding of the ET mechanisms of dihydrochalcones. In addition, some multiple mechanism-based antioxidant assays were also conducted in the study, such as 2,2′-azino-bis(3-ethylbenzo-thiazoline-6-sulfonic acid) (ABTS^+^•)-scavenging assay, •O_2_^−^ -scavenging assay, and 1,1-diphenyl-2-picryl-hydrazl radical (DPPH•)-scavenging assay. DPPH•-scavenging, however, was further investigated for the radical adduct formation (RAF) pathway using ultra-performance liquid chromatography coupled with electrospray ionization quadrupole time-of-flight tandem mass spectrometry (UPLC−ESI−Q−TOF−MS/MS) technology.

In summary, this study, based on five similar dihydrochalcones, will provide an understanding of antioxidant pathways and the role of the various substituents in the dihydrochalcone family of antioxidants.

## 2. Results and Discussion

ET is an important antioxidant pathway of phenolic compounds [16,17]. Through this pathway, electrons are lost from the (phenolic) antioxidant and react with reactive oxygen species (ROS) or reactive nitrogen species (RNS) [13]. Under normal conditions, ROS and RNS can induce stem cell proliferation and differentiation [18,19]. Under pathological conditions, excessive ROS or RNS can cause oxidative damage to biomolecules and worsen the oxidative status of cells [20,21]. Because ET can effectively improve cellular oxidative status via inhibition of the generation of ROS or RNS, it thus plays a critical role in the improvement of (stem) cell quality [22,23].

To explore the ET potential of the five dihydrochalcones, they were determined using the FRAP assay [24]. As shown in Suppl. 1, the FRAP activities of phloretin, neohesperidin dihydrochalcone, and the positive control (Trolox) increased in a dose-dependent manner, whereas those of the others moderately increased with increasing concentration, suggesting that the five dihydrochalcones have greater ET potential.

The 50% radical inhibition (IC_50_) value in micromoles was obtained from the dose–response curves shown in Suppl. 1. The IC_50_ values with different letters (a, b, c, d, or e) in the same column indicate significant (*p* < 0.05) differences between the five dihydrochalcones and the positive control (Trolox).

To explore the ET potential further, the ABTS•^+^-scavenging assay was used. ABTS•^+^-scavenging is essentially an ET reaction [25] because ABTS•^+^ tends to gain an electron from phenolic antioxidants to neutralize the positive charge and form a stable ABTS molecule [26]. As illustrated in Suppl. 1, the five dihydrochalcones showed increased ABTS•^+^-scavenging activities depending on the dosage, meaning that five dihydrochalcones undergo ET reactions to scavenge radicals.

**Figure 2:**
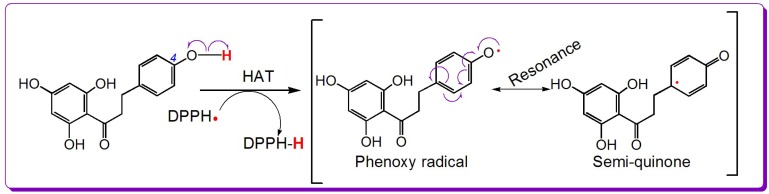
The proposed reaction of the 1,1-diphenyl-2-picryl-hydrazl (DPPH•) radical with the 4-OH group of phloretin.

Besides ET, hydrogen atom transfer (HAT) frequently takes place during the antioxidant reactions of phenolics [27]. Usually, the DPPH•-scavenging model is used to explore HAT ability because HAT is a necessary reaction in the DPPH•-scavenging process [28]. As shown in Suppl. 1, the DPPH•-scavenging activities of the five dihydrochalcones increased in a dose-dependent manner, suggesting that they could also scavenge radicals via HAT. For example, the 4-OH of phloretin can prompt HAT to scavenge DPPH•. Based on the previous literature [20,29], the HAT reaction is shown in Figure 2.

However, ABTS•^+^ and DPPH• are chemical radicals and are not found naturally in cells. The radicals occurring in cells are mainly ROS and RNS, such as •O_2_^−^, •OH, •NO, and •ONOO^−^ [30]. Because the •O_2_^−^ radical anion can be produced from O_2_ and further transferred to •OH and H_2_O_2_ molecules, •O_2_^−^ is, thus, regarded as an important ROS [29,31]. In this study, •O_2_^−^ was successfully scavenged by the five dihydrochalcones in a concentration-dependent manner (Suppl. 1). In other words, the five dihydrochalcones had ROS-scavenging ability. In fact, •O_2_^−^ scavenging has previously been suggested to be involved in ET and HAT pathways [32].

**Figure 3:**
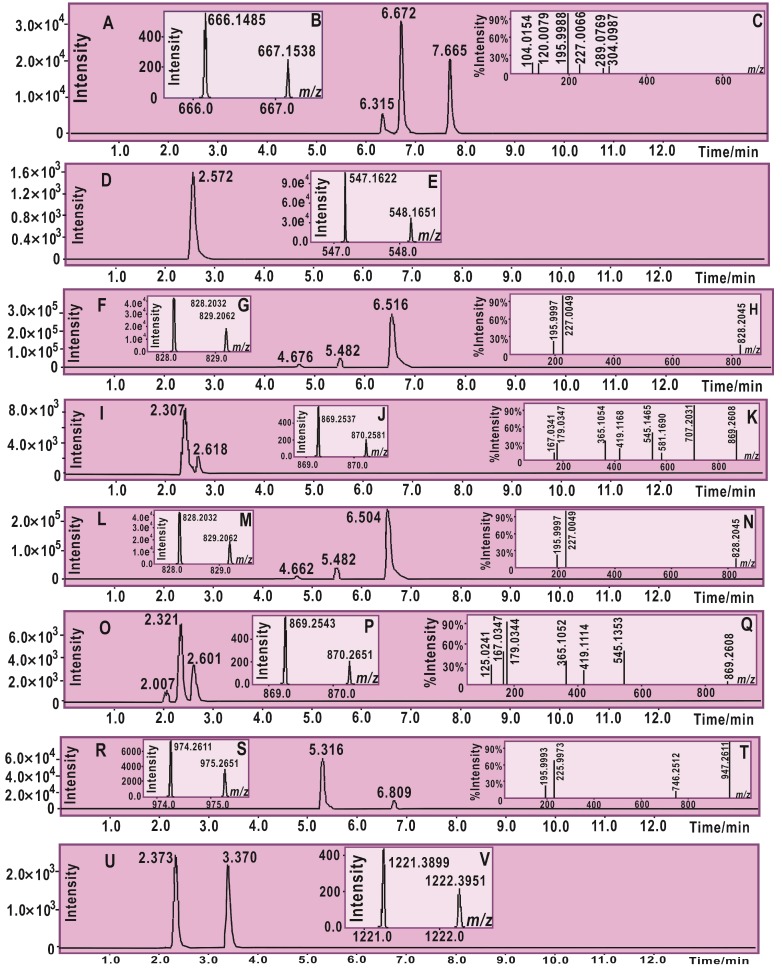
The main results of ultra-performance liquid chromatography coupled with electrospray ionization quadrupole time-of-flight tandem mass spectrometry (UPLC−ESI−Q−TOF−MS/MS) analysis for radical adduct formation (RAF) products: (**A**) chromatogram of RAF products of phloretin with DPPH• after the formula [C_33_H_25_N_5_O_11_-H]^−^ had been extracted; (**B**) primary MS spectra of phloretin-DPPH adducts (*m*/*z* 666–667 for their molecular ion peaks); (**C**) MS/MS spectra of phloretin-DPPH adducts (*m*/*z* 196, 227 for fragments from the DPPH moiety, Suppl. 2); (**D**) chromatogram of possible dimeric products of phloretin after the formula [C_30_H_26_O_10_-H]^−^ had been extracted; (**E**) primary MS spectra of the phloretin-phloretin dimer (*m*/*z* 547–548 for their molecular ion peaks); (**F**) chromatogram of RAF products of trilobatin with DPPH• after the formula [C_39_H_35_N_5_O_16_-H]^−^ had been extracted; (**G**) primary MS spectra of trilobatin-DPPH adducts (*m*/*z* 828–829 for their molecular ion peaks); (**H**) MS/MS spectra of trilobatin-DPPH adducts (*m*/*z* 196, 227 for fragments from DPPH moiety, Suppl. 2); (**I**) chromatogram of possible dimeric products of trilobatin after the formula [C_42_H_46_O_20_-H]^−^ had been extracted; (**J**) primary MS spectra of trilobatin-trilobatin dimers (*m*/*z* 869–870 for their molecular ion peaks); (**K**) MS/MS spectra of RAF product of trilobatin-trilobatin dimers (*m*/*z* 707 for fragments losing a glucose residue); (**L**) chromatogram of RAF products of phloridzin with DPPH• after the formula [C_39_H_35_N_5_O_16_-H]^−^ had been extracted; (**M**) Primary MS spectra of phloridzin-DPPH adducts (*m*/*z* 828–829 for their molecular ion peaks); (**N**) MS/MS spectra of phloridzin-DPPH adducts (*m*/*z* 196 and 227 for fragments from DPPH moiety, Suppl. 2); (**O**) chromatogram of possible dimeric products of phloridzin after the formula [C_42_H_46_O_20_-H]^−^ had been extracted; (**P**) primary MS spectra of phloridzin-phloridzin dimers (*m*/*z* 869–870 for their molecular ion peaks); (**Q**) MS/MS spectra of the phloridzin-phloridzin dimers (*m*/*z* 167, 179 for loss of glucose residue); (**R**) chromatogram of RAF product of naringin dihydrochalcone with DPPH• after the formula [C_45_H_45_N_5_O_20_-H]^−^ had been extracted; (**S**) primary MS spectra of naringin dihydrochalcone-DPPH adducts (*m*/*z* 975–975 for their molecular ion peaks); (**T**) MS/MS spectra of naringin dihydrochalcone-DPPH adducts (*m*/*z* 196 and 227 for fragments from the DPPH moiety, Suppl. 2); (**U**) chromatogram of possible dimeric products of neohesperidin dihydrochalcone after the formula [C_56_H_70_O_30_-H]^−^ had been extracted; (**V**) primary MS spectra of neohesperidin dihydrochalcone-neohesperidin dihydrochalcone dimers (*m*/*z* 1221–1222 for their molecular ion peaks). (Note: each of the peaks in one chromatographic diagram gave similar primary MS spectra and MS/MS spectra. For example, each of three peaks of phloretin-DPPH adducts (RT = 6.315, 6.672, 7.665 min in (**A**)) similarly produced primary MS spectra (**B**) and MS/MS spectra (**C**)). The original spectra are listed in Suppl. 3.

During the ROS-scavenging process of phenolic compounds, radical adduct formation (RAF) may also play a role [33]. To provide evidence for RAF, each of the five dihydrochalcones was mixed with the DPPH• radical in methanol solution. The product mixture was further analyzed using ultra-performance liquid chromatography coupled with electrospray ionization quadrupole time-of-flight tandem mass spectrometry (UPLC−ESI−Q−TOF−MS/MS). As shown in Figure 3 and the MS spectra elucidation (Suppl. 2), there were RAF products in the mixture of dihydrochalcone with DPPH•; The RAF products however comprised dihydrochalcone-DPPH adduct and dihydrochalcone-dihydrochalcone dimer.

Thus, it is clear that five dihydrochalcones have antioxidant potential, and their antioxidant pathways may involve ET, HAT, and RAF. However, concerning the chemistry, RAF is dissimilar to ET and HAT. Both ET and HAT are based on redox reactions: phenolic antioxidant transfer of an electron (***e***) or hydrogen atom (H•) to radicals (e.g., ABTS•^+^, DPPH•, and •O_2_^−^) or the loss of an electron or hydrogen atom from a phenolic antioxidant to form its oxidized product (e.g., phenoxy radical or semiquinone). Because of conjugation, the phenoxy radical or semiquinone is somewhat stable. Such stability could be responsible for the ET (or HAT) reactivity of the dihydrochalcones towards free radicals. RAF reactions are based on the formation of covalent bonds. Thus, they are not redox reactions [34]. In summary, the antioxidant pathways of the five dihydrochalcones can be divided into two main types: redox-based antioxidant pathways (i.e., ET and HAT) and covalent-based antioxidant pathway (i.e., RAF).

In the four redox-based antioxidant assays (i.e., FRAP, ABTS•^+^-scavenging, DPPH• scavenging, and •O_2_^−^ scavenging assays), the five dihydrochalcones showed different antioxidant activities. For comparison, the five dihydrochalcones were combined into five pairs.

The first pair is phloretin and phloridzin. As shown in Figure 1, the sole difference between phloretin and phloridzin is at the 2′-position: phloretin bears a 2′-OH, whereas in phloridzin the 2′-OH is glycosylated by *β*-d-glucoside. The IC_50_ values revealed that, in the four redox-based antioxidant assays, phloretin showed higher antioxidant capacity than its glucoside phloridzin. This result strongly indicates that the glycosylation of the phenolic –OH reduces the redox-based antioxidant potential (especially ET and HAT). This is because the glycosylation of –OH reduces the number of phenolic –OH groups, and phenolic –OH is the source of the antioxidant capacity.

The second pair studied is phloretin and trilobatin. This pair is similar to the first pair because trilobatin is also a glycoside of phloretin. As listed in Table 1, trilobatin had higher IC_50_ values than those of phloretin in the four redox-based antioxidant assays. The findings further support the conclusions obtained from the first pair; that is, the glycosylation of phenolic –OH reduces the antioxidant potential of dihydrochalcones. In fact, similar glycosylation activity toward antioxidant ability has also been observed in flavonoids, e.g., dihydromyricetin [27], baicalein, and baicalin [35].

The third pair is trilobatin and naringin dihydrochalcone. As shown in Figure 1, naringin dihydrochalcone is the rhamnose glycoside of trilobatin. The glycoside is at 2″-position where there is an alcoholic –OH (rather than a phenolic –OH), and, thus, the number of phenolic –OH groups is not reduced. Nevertheless, the IC_50_ values in Table 1 indicate that naringin dihydrochalcone always exhibited weaker antioxidant activity than trilobatin in the four redox-based antioxidant assays. This suggests that glycosylation itself can decrease the antioxidant capacity even if the number of phenolic –OH groups is not reduced.

**Figure 4:**
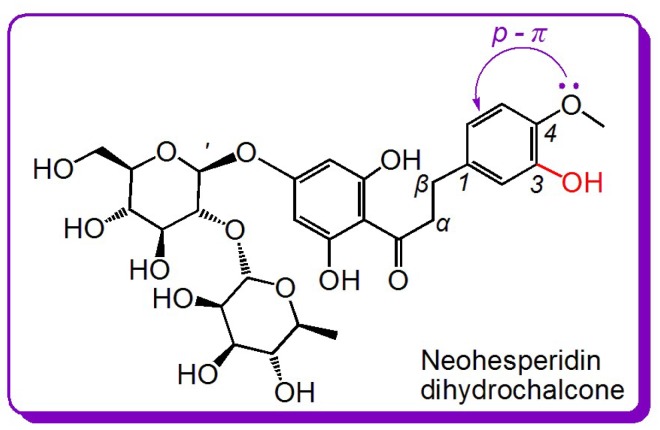
The electron donating effect of –OCH_3_ towards the benzo ring via *p*-π conjugation in neohesperidin dihydrochalcone.

The fourth pair is neohesperidin dihydrochalcone and naringin dihydrochalcone. As shown in Figure 1, the difference between these two compounds is an extra –OCH_3_ group in neohesperidin dihydrochalcone. The IC_50_ values in Table 1 reveal that, in the four redox-based antioxidant assays, neohesperidin dihydrochalcone exhibited higher levels than naringin dihydrochalcone. The enhancement of –OCH_3_ group can be attributed to the electron-donating *p*-π conjugation of the –OCH_3_ group to the benzo ring. The enrichment of π-electrons correspondingly increases the antioxidant capacity of the *ortho*-OH group (Figure 4). Notably, the substituent positions between the two compounds are different: naringin dihydrochalcone bears a 4-OH group, whereas neohesperidin dihydrochalcone contains 3-OH and 4-OCH_3_ groups. When the 3-OH or 4-OH group is oxidized to a phenoxy radical or semiquinone, the saturated alkyl at 1-position has not helped to stabilize the phenoxy radical or semiquinone [36]. Thus, the 4-OH group is identical to the 3-OH group, and the antioxidant-promoting effect of the –OCH_3_ group is definitive. This is further supported by the antioxidant structure–activity analysis of 4-hydroxybenzoic acid and 4-hydroxy-3-methoxy benzoic acid (vanillic acid) [13].

The fifth pair is phloridzin and trilobatin. As shown in Figure 1, the sole difference between phloridzin and trilobatin is the position of the glycoside: phloridzin at 2′-OH and trilobatin at 4′-OH. As shown in Table 1, trilobatin always exhibited greater antioxidant ability compared to phloridzin in the four redox-based antioxidant assays. In the ABTS^+^·-scavenging and DPPH•-scavenging assays, trilobatin has three-times higher scavenging ability than its isomer phloridzin. Thus, the glycosylation position has a significant effect on the antioxidant capacities of dihydrochalcones. This interesting effect may be associated with two issues, i.e., the degree of resonance and the intramolecular hydrogen bond (IHB).

**Figure 5:**
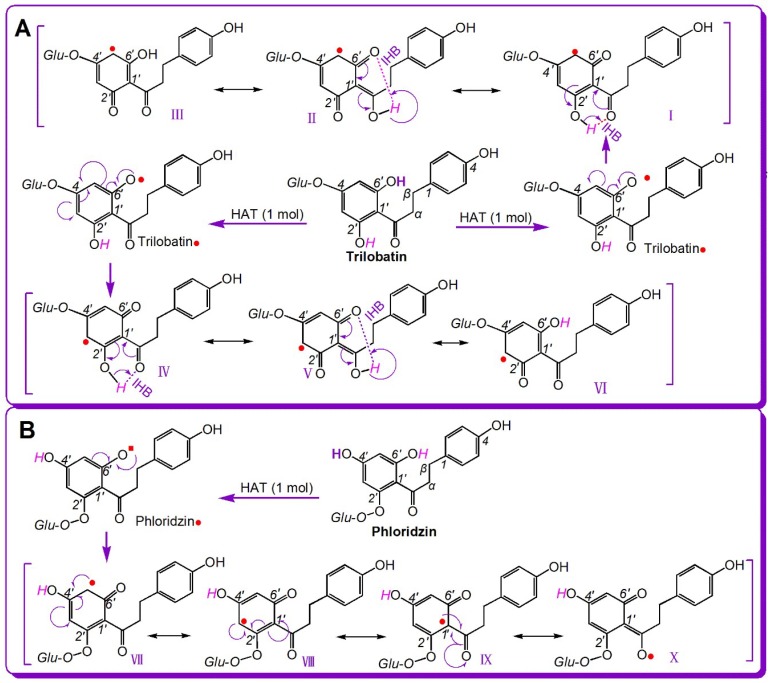
The resonance formula of trilobatin• (**A**) and phloridzin• (**B**).

Seemingly, the difference between phloridzin and trilobatin lies in glycosylation position. However, the different glycosylation position results in the formation of different chemical moieties: 2′,6′-di-OHs in phloridzin and 4′,6′-di-OHs in trilobatin (Figure 1). Both compounds have a 6′-OH group, and the 6′-OH group undergoes HAT to form 6′-phenoxyl radicals (i.e., phloridzin• and trilobatin•, respectively, Figure 5). Trilobatin• has six resonance forms (I–VI) (Figure 5A). In comparison, phloridzin• has only four resonance forms (VII–X) (Figure 5B). Thus, the HAT product trilobatin• is more stable than phloridzin•, and trilobatin is more active than phloridzin.

As shown in Figure 5, trilobatin• has more resonance forms than phloridzin• because the H atom at the 2′-OH in trilobatin• participates in resonance via keto–enol tautomerism (Figure 5A(IV–VII)). The keto–enol tautomerism was facilitated by the IHB between the 2′-OH group and adjacent keto group (Figure 5A(VI)). In contrast, phloridzin• has no IHB or keto–enol tautomerism (Figure 5B). Thus, for the dihydrochalcones, the 2′,6′-di-OH moiety results in a more powerful antioxidant effect than the 4′,6′-di-OH moiety. Our analysis based on resonance theory further explains the previous finding that IHBs play a beneficial role in the antioxidant effect of myrigalone B (2′,6′-dihydroxy-4′-methoxy-3′,5′-dimethyl-dihydrochalcone) [37].

The role of IHBs in the dihydrochalcone family is different from that in the flavonoid family. In flavonoids, IHBs usually reduce the antioxidant capacity. For example, 5-hydroxyflavone is half as active as 6-hydroxyflavone [13,37] because the IHB hinders dissociation from the phenolic moiety. Furthermore, the 3,5-di-OH flavonoid shows little increase in antioxidant capacity compared to other flavonoids, despite the 3,5-di-OH flavonoid having a similar extensive resonance to 2′,6′-di-OH dihydrochalcone [38]. This is because that flavonoid lacks a freely-rotating σ-bond. The IHBs in flavonoids can only limit H dissociation and cannot stabilize phenoxy radicals or semiquinones. However, in dihydrochalcones (especially 2′,6′-dihydroxy dihydrochalcone), the IHB can stabilize the phenoxyl radical (or semiquinone) via resonance. Thus, IHBs enhance the antioxidant capacity of the dihydrochalcones.

**Figure 6:**
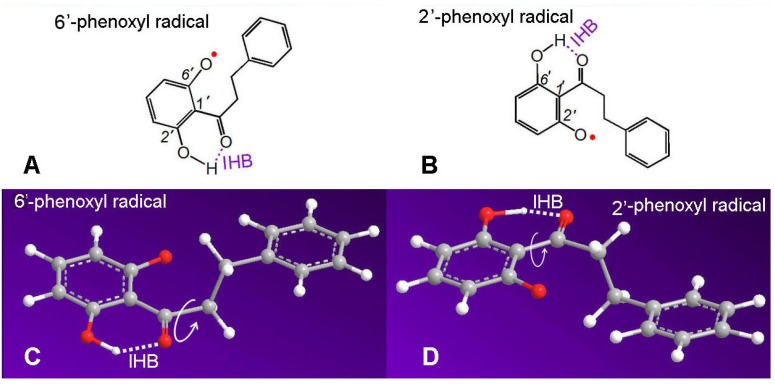
The intramolecular hydrogen bond (IHB) between 2′-OH (or 6′-OH) and adjacent keto group of the phenoxyl radical in 2′,6′-dihydroxy dihydrochalcone.

As shown in Figure 6, if the 2′,6′-dihydroxy dihydrochalcone donates an H atom, and it can form 6′-phenoxyl or 2′-phenoxyl radicals. The 6′-phenoxyl radical can form an IHB between 2′-OH and the adjacent keto group, whereas the 2′-phenoxyl radical can form an IHB between 6′-OH and the adjacent keto group (Figure 6A,B). The reason why the keto group can form an IHB with either the 2′-OH or 6′-OH group is that the σ-bond linking the 1′-carbon and adjacent keto group can freely rotate (Figure 6C,D). Thus, both the 6′-phenoxyl radical and 2′-phenoxyl radicals are stable. Undoubtedly, IHBs play a stabilizing role in this process. The stability of the phenoxyl radical could be responsible for the strong antioxidant capacity (especially the HAT capacity) of 2′,6′-dihydroxy dihydrochalcone. However, it must be noted that resonance (including keto–enol tautomerism) occasionally takes place after phenoxyl radical generation, but a stable phenolic molecule cannot be converted to a ketone via tautomerism because there is a complete phenolic moiety containing a benzo ring.

As mentioned above, RAF may play a role in the antioxidant action of the five dihydrochalcones [39]. Nevertheless, further analysis indicated that the RAF activity differs between the five dihydrochalcones. As illustrated in Figure 3, phloretin, phloridzin, and trilobatin gave rise to two kinds of RAF products, i.e., dihydrochalcone-DPPH adduct, and dihydrochalcone-dihydrochalcone dimer, whereas neohesperidin dihydrochalcone and naringin dihydrochalcone only produced either dihydrochalcone-DPPH adduct or dihydrochalcone-dihydrochalcone dimer. Naringin dihydrochalcone gave a naringin dihydrochalcone-DPPH adduct, whereas neohesperidin dihydrochalcone gave neohesperidin dihydrochalcone-neohesperidin dihydrochalcone dimers. These results suggest the weaker RAF potential of neohesperidin dihydrochalcone and naringin dihydrochalcone. The reason why the latter two exhibited weaker RAF potential than the former three is possibly because the latter two have two sugar residues, resulting in steric hindrance preventing the RAF reaction. Our assumption is further confirmed by the evidence from phloretin. Phloretin without a sugar residue has the least steric hindrance, yielding three phloretin-DPPH RAF products (retention time (R.T.)) = 6.315, 6.672, and 7.665 min, Figure 3). However, the multiple products may originate from different locations of the covalent bond formed after radical attack [40].

## 3. Materials and Methods 

### 3.1. Chemicals

Phloretin (CAS 60-82-2, C_15_H_14_O_5_, M.W. 274.2, purity 98%, off-white powder, Suppl. 4), phloridzin (CAS 60-81-1, C_21_H_24_O_10_, M.W. 436.4, purity 98%, off-white powder, Suppl. 4), trilobatin (CAS 4192-90-9, C_21_H_24_O_10_, M.W. 436.4, purity 98%, pale yellow powder, Suppl. 4), neohesperidin dihydrochalcone (CAS 20702-77-6, C_28_H_36_O_15_, M.W. 612.2, purity 98%, off-white powder, Suppl. 4), and naringin dihydrochalcone (CAS 18916-17-1, C_27_H_34_O_14_, M.W. 582.6, purity 98%, off-white powder, Suppl. 4) were obtained from Chengdu Biopurify Phytochemicals Ltd. (Chengdu, China). 1,1-Diphenyl-2-picrylhydrazyl radical (DPPH•), (±)-6-hydroxyl-2,5,7,8-tetramethlychromane-2-carboxylic acid (Trolox), 2,4,6-tripyridyltriazine (TPTZ), and pyrogallol were purchased from Sigma-Aldrich Shanghai Trading Co. (Shanghai, China). (NH_4_)_2_ABTS [2,2′-azino-bis(3-ethylbenzo-thiazoline-6-sulfonic acid) diammonium salt] was obtained from the Amresco Chemical Co. (Solon, OH, USA). Methanol and water were of high-performance liquid chromatography (HPLC) grade. All other reagents used in this study were purchased as analytical grade from the Guangzhou Chemical Reagent Factory (Guangzhou, China).

### 3.2. Ferric-Reducing Antioxidant Power (FRAP) Assay (Fe^3+^-Reducing)

The Fe^3+^-reducing assay was established by Benzie and Strain and is known as the FRAP assay [16]. The experimental protocol for this assay has been described previously [35]. Briefly, the FRAP reagent was prepared freshly by mixing 10 mM TPTZ, 20 mM FeCl_3_, and 0.25 M acetate buffer at a ratio of 1:1:10 at pH 3.6. The test sample (*x* = 2–10 μL, 1 mg/mL) was added to (20–*x*) μL of 95% ethanol followed by 80 μL of the FRAP reagent. After a 30-min incubation at 30 °C, the absorbance was measured at 595 nm using a microplate reader (Multiskan FC, Thermo Scientific, Shanghai, China). The relative reducing power of the sample was calculated as:

$$Relative\;reducing\;effect\;\%=\frac{A-A_{\min}}{A_{\max}-A_{\min}}\times 100\%$$

where *A_max_* is the maximum absorbance of the reaction mixture with sample, *A_min_* is the minimum absorbance in the test, and *A* is the absorbance of the sample.

### 3.3. ABTS^+^•-Scavenging Assay

The ABTS^+^•-scavenging activity was evaluated according to the reported method [41]. The ABTS^+^• radical was produced by mixing 0.2 mL of ABTS diammonium salt (7.4 mmol/L) with 0.2 mL of potassium persulfate (2.6 mmol/L). The mixture was kept in the dark at room temperature for 12 h to allow completion of radical generation before dilution with distilled water (at a ratio of approximately 1:20) so that its absorbance at 734 nm was 0.35 ± 0.01, determined using the aforementioned microplate reader. To determine the scavenging activity, the test sample (*x* = 2–10 μL, 0.25 mg/mL) was added to (20–*x*) μL of distilled water followed by 80 μL of ABTS^+^• reagent, and the absorbance at 734 nm was measured 3 min after the initial mixing using distilled water as the blank. The percentage of ABTS^+^•-scavenging activity was calculated as

$$Scavenging\;\%\;=\;\frac{A_{0}-A}{A_{0}}\;\times \;100\%$$

where *A*_0_ is the absorbance of the control without the sample at 734 nm, and *A* is the absorbance at 734 nm of the reaction mixture with the sample.

### 3.4. DPPH•-Scavenging Assay

The DPPH• radical-scavenging assay has been described previously [37]. Briefly, 80 μL DPPH•-methanolic solution (0.1 mol/L) was mixed with a 1 mg/mL methanolic solution of the sample (2–10 μL). The mixture was maintained at room temperature for 30 min, and the absorbance was measured at 519 nm on a microplate reader. The percentage of DPPH•-scavenging activity was calculated based on the formula presented in Section 3.2.

### 3.5. Superoxide Anion Radical (•O_2_^–^)-Scavenging Assay 

The superoxide anion radical (•O_2_^–^)-scavenging assay method was developed in our laboratory [42]. Briefly, the sample was dissolved in ethanol at 5 mg/mL. The sample solution (*x* μL, where *x* = 2–10 μL) was mixed with (980–*x*) μL Tris-HCl buffer (0.05 M, pH 7.4) containing the disodium salt of ethylenediaminetetraacetic acid (1 mM). After 20 μL pyrogallol (60 mM in 1 mM HCl) had been added, the mixture was shaken at room temperature immediately. The absorbance of the mixture at 325 nm was measured (Unico 2100, Shanghai, China) against Tris-HCl buffer as a blank every 30 s for 5 min. The •O_2_^–^ scavenging ability was calculated as

$$Inhibition\;\%=\frac{\left(\frac{\Delta A_{325nm,control}}{T}\right)-\left(\frac{\Delta A_{325nm}{}_{,sample}}{T}\right)}{\left(\frac{\Delta A_{325nm}{}_{,control}}{T}\right)}\times 100\%$$

where Δ*A_325nm,control_* is the increase in *A*_325nm_ of the mixture without the sample, and Δ*A_325nm,sample_* is that with the sample; T = 5 min.

### 3.6. Ultra-Performance Liquid Chromatography Coupled with Electrospray Ionization Quadrupole Time-of-Flight Tandem Mass Spectrometry (UPLC−ESI−Q−TOF−MS/MS) Analysis of Dihydrochalcone Reaction Product with DPPH•

UPLC−ESI−Q−TOF−MS/MS spectra of the reaction products of DPPH• with the dihydrochalcone phenolic components were obtained according to our previously described method [43]. The methanolic solutions of phenolic components were mixed with a solution of DPPH• radicals in methanol at a molar ratio of 1:2, and the resulting mixtures were incubated for 24 h at room temperature. The product mixtures were filtered through a 0.22-μm filter and measured using a UPLC–ESI–Q–TOF–MS/MS system equipped with a C_18_ column (2.0 mm i.d. × 100 mm, 2.2 μm, Shimadzu Co., Kyoto, Japan). The mobile phase was used for elution and consisted of a mixture of methanol (phase A) and water (phase B). The product mixture column was eluted at a flow rate of 0.3 mL/min with the following gradient elution program: 0–10 min, 60–100% A; 10–15 min, 100% A. The sample injection volume was set at 1 μL to separate the components, and the column temperature was 40 °C. The Q–TOF–MS/MS analysis was conducted on a Triple TOF 5600+ mass spectrometer (AB SCIEX, Framingham, MA, USA) equipped with an ESI source, which was run in the negative ionization mode. The scan range was set at 50–1600 Da. The system was run with the following parameters: ion spray voltage, −4500 V; ion source heater, 550 °C; curtain gas (CUR, N_2_), 30 psi; nebulizing gas (GS1, air), 50 psi; and TurboIonSpray (TIS) gas (GS2, air), 50 psi. The declustering potential (DP) was set at −100 V, and the collision energy (CE) was set at −40 V with a collision energy spread (CES) of 20 V. The RAF products were quantified by the removing the corresponding formula (e.g., [C_33_H_25_N_5_O_11_-H]^−^ for phloretin-DPPH and [C_30_H_26_O_10_-H]^−^ for the phloretin-phloretin dimer) from the total ion chromatogram and integrating the corresponding peaks.

### 3.7. Statistical Analysis

Each experiment was performed in triplicate and the data were recorded as the mean ± standard deviation (SD). The dose-response curves were plotted using Origin 6.0 Professional (OriginLab, Northampton, MA, USA). The IC_50_ value was defined as the final concentration of 50% radical inhibition (or relative reducing power). The IC_50_ was calculated by linear regression analysis and expressed as the mean ± SD (*n* = 3) [39]. The linear regression was analyzed using Origin 6.0 Professional. Statistical comparisons were made by one-way analysis of variance (ANOVA) to detect significant differences using SPSS 13.0 (SPSS Inc., Chicago, IL, USA) for Microsoft Windows. A value of *p* < 0.05 was considered to be statistically significant.

## 4. Conclusions

The five dihydrochalcone antioxidants may possess ET, HAT, and RAF activity. Methoxylation at the *ortho*-OH can greatly enhance the ET potential via *p*-π conjugation. The 2′,6′-di-OH moiety in dihydrochalcone has greater ET and HAT activities than the 2′,4′-di-OH moiety owing to the wide resonance with the adjacent keto group. Glycosylation can not only reduce the number of phenolic hydroxyl groups (thus lowering the antioxidant activity) but also reduce the RAF potential via steric hindrance.

## Figures and Tables

**Table 1:** The IC_50_ values of five dihydrochalcones in various antioxidant assays.

Analysis	FRAP μM	ABTS^+^• Scavenging μM	DPPH• Scavenging μM	•O_2_^−^ Scavenging μM
Phloretin	666.4 ± 90.4 ^b^	4.3 ± 1.8 ^a^	63.5 ± 4.6 ^b^	108.4 ± 6.4 ^a^
Phloridzin	2956.51 385.9 ^d^	65.8 ± 2.0 ^e^	561.0 ± 34.0 ^e^	385.2 ± 47.8 ^c^
Trilobatin	2275.3 ± 158.5 ^c^	17.4 ± 0.7 ^b^	164.0 ± 1.4 ^c^	270.6 ± 25.0 ^b^
Naringin dihydrochalcone	2650.7 ± 61.7 ^d^	24.0 ± 2.4 ^c^	318.9 ± 19.6 ^d^	322.8 ± 19.9 ^c^
Neohesperidin dihydrochalcone	93.7 ± 11.4 ^a^	20.5 ± 0.2 ^b^	58.3 ± 7.9 ^b^	56.0 ± 1.7 ^a^
Trolox	136.6 ± 15.6 ^a^	33.0 ± 3.4 ^d^	13.4 ± 0.4 ^a^	953.4 ± 57.1 ^d^

The IC_50_ value was expressed as the mean ±SD (*n* = 3). The IC_50_ values with different superscripts (a, b, c, d, or e) in the same column are significantly different (*p* < 0.05). Trolox is the positive control.

## Supplementary Materials

The following are available online. Suppl. 1. Dose-response curves, Suppl. 2. DPPH MS analysis elucidation, Suppl. 3. Original data from high-performance liquid chromatography-mass spectrometry (HPLC-MS) analysis, Suppl. 4. Appearances and analysis certificates and of five dihydrochalcones.

## Abbreviations

The following abbreviations are used in this manuscript:

ABTS	2,2′-azino-bis(3-ethylbenzo-thiazoline-6-sulfonic acid diammonium salt)
DPPH•	1,1-diphenyl-2-picryl-hydrazyl radical
ET	electron transfer
FRAP	ferric reducing antioxidant power
HAT	hydrogen atom transfer
ROS	reactive oxygen species
RAF	radical adduct formation
SD	standard deviation
TPTZ	2,4,6-tris(2-pyridyl-s-triazine)
Trolox	(±)-6-hydroxyl-2,5,7,8-tetramethlychromane-2-carboxylic acid
