# Supplementary material for: Antioxidant Structure–Activity Relationship Analysis of Five Dihydrochalcones
Source: Molecules. 2018 May 12;23(5):1162. doi: 10.3390/molecules23051162 (PMC6100071; doi:10.3390/molecules23051162)
Supplement: Supplementary file 1 [file molecules-23-01162-s001.zip › Supples/Suppl 3 Original data of HPLC-MS.docx]

**Supplementary Material 3**

**Antioxidant Structure–Activity Relationship Analysis of Five Dihydrochalcones**

**Xican Li ^1, 2, *^, Ban Chen ^1, 2^, Hong Xie ^1, 2^, Yuhua He ^1^**, **Dewei Zhong ^1^, and Dongfeng Chen ^3, 4, *^**

^1^ School of Chinese Herbal Medicine, Guangzhou University of Chinese Medicine, Waihuan East Road No. 232, Guangzhou Higher Education Mega Center, Guangzhou 510006, China. [imchenban@foxmail.com](mailto:imchenban@foxmail.com) (B.C.); [xiehongxh1@163.com](mailto:xiehongxh1@163.com) (H.X.); [505780526@qq.com](mailto:505780526@qq.com) (Y.H.); [393916211@qq.com](mailto:393916211@qq.com) (D.Z.).

^2^ Innovative Research & Development Laboratory of TCM, Guangzhou University of Chinese Medicine Waihuan East Road No. 232, Guangzhou Higher Education Mega Center, Guangzhou 510006, China.

^3^ School of Basic Medical Science, Guangzhou University of Chinese Medicine, Waihuan East Road No. 232, Guangzhou Higher Education Mega Center, Guangzhou 510006, China.

^4^ The Research Center of Basic Integrative Medicine, Guangzhou University of Chinese Medicine, Waihuan East Road No. 232, Guangzhou Higher Education Mega Center, Guangzhou 510006, China.

^*^ Corresponding author. E-mail: [lixican@126. com](mailto:lixican@126.com)(X.L.); lixc@gzucm.edu.cn(X.L.); chen888@gzucm.edu.cn(D.C.); Tel.: +86-203-935-8076 (X.L.)

**1. chromatogram of RAF products of phloretin with DPPH• when the formula [C_33_H_25_N_5_O_11_-H]^-^ was extracted.**


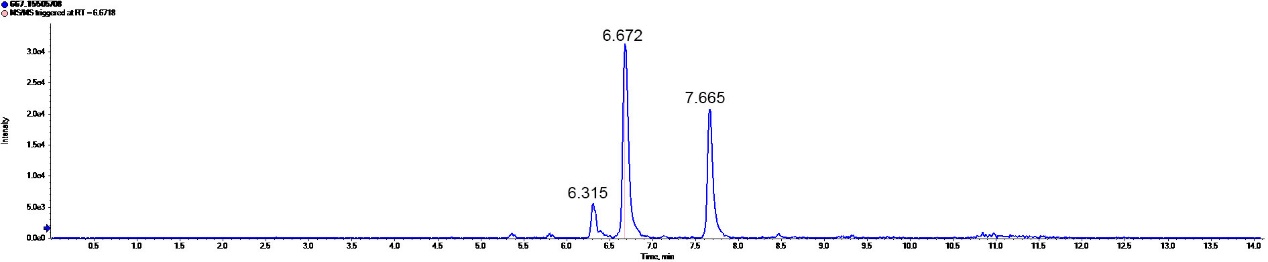


**Primary MS spectra of Rt=6.315 min**

**MS/MS spectra of** **Rt=6.315 min**

**Rt=6.672 min**

**Primary MS spectra of Rt=6.672 min**

**MS/MS spectra of** **Rt=6.672 min**

**Rt=7.665 min**

**Primary MS spectra of Rt=7.665 min**

**MS/MS spectra of Rt=7.665 min**

**2. chromatogram of RAF products of trilobatin with DPPH• when the formula [C_39_H_35_N_5_O_16_-H]^-^ was extracted.**


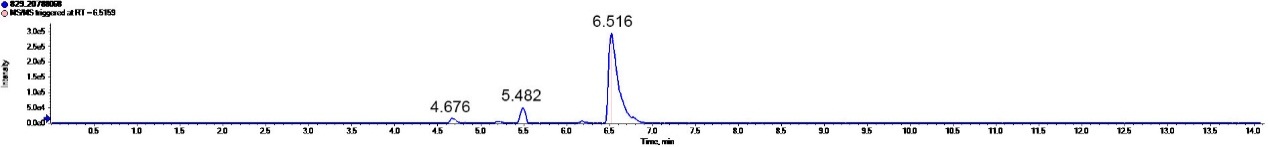


**Rt=4.676 min**

**Primary MS spectra of Rt=4.676 min**

**MS/MS spectra of Rt=4.676 min**

**Rt=5.482 min**

**Primary MS spectra of Rt=5.482 min**

**MS/MS spectra of Rt=4.676 min**

**Rt=6.516 min**

**Primary MS spectra of Rt=6.516 min**

**MS/MS spectra of Rt=6.516 min**

**3. chromatogram of possible dimeric products of trilobatin when the formula [C_42_H_46_O_20_-H]- was extracted.**

**
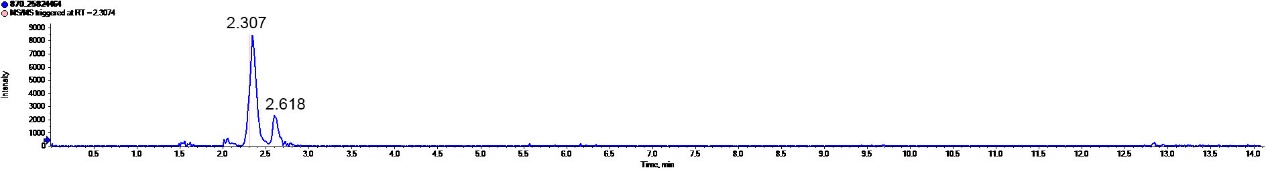
**

**Rt=2.307 min**

**Primary MS spectra of Rt=2.307 min**

**MS/MS spectra of Rt=2.307 min**

**Rt=2.618 min**

**Primary MS spectra of Rt=2.618 min**

**4. chromatogram of RAF products of phloridzin with DPPH• when the formula [C_39_H_35_N_5_O_16_-H]^-^ was extracted.**

**
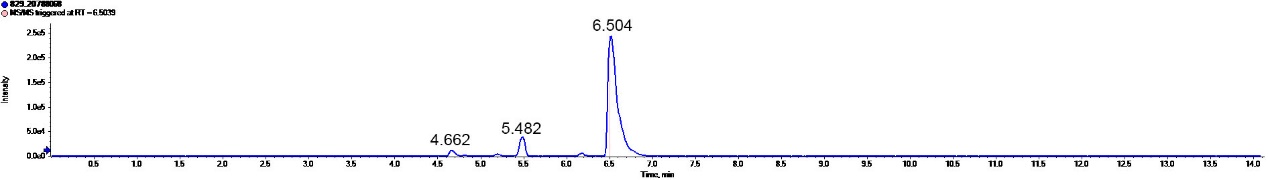
**

**Rt=4.662 min**

**Primary MS spectra of Rt=4.662 min**

**MS/MS spectra of Rt=4.662 min**

**Rt=5.482 min**

**Primary MS spectra of Rt=5.482 min**

**MS/MS spectra of Rt=5.482 min**

**Rt=6.504 min**

**Primary MS spectra of Rt=6.504 min**

**MS/MS spectra of Rt=6.504 min**

**5. chromatogram of possible dimeric products of phloridzin when the formula [C_42_H_46_O_20_-H]^-^ was extracted.**

**
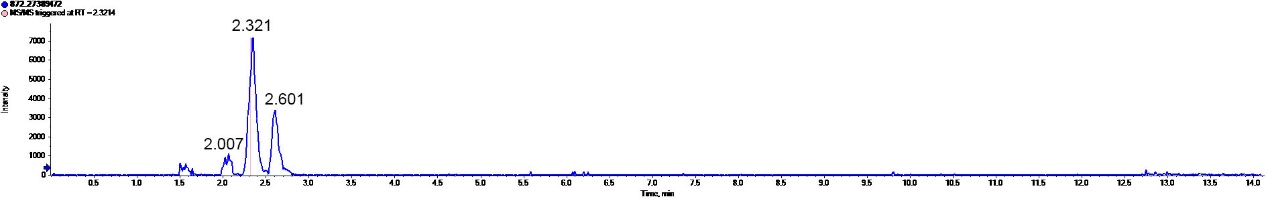
**

**Rt=2.007 min**

**Primary MS spectra of Rt=2.007 min**

**MS/MS spectra of Rt=2.007 min**

**Rt=2.321 min**

**Primary MS spectra of Rt=2.321 min**

**MS/MS spectra of Rt=2.321 min**

**Rt=2.601 min**

**Primary MS spectra of Rt=2.601 min**

**6. chromatogram of RAF product of naringin dihydrochalcone with DPPH• when the formula [C_45_H_45_N_5_O_20_-H]^-^ was extracted.**

**
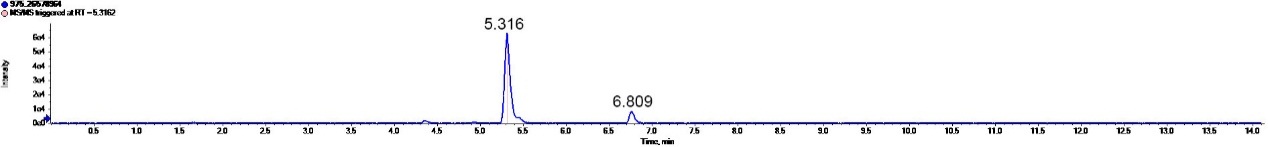
**

**Rt=5.316 min**

**Primary MS spectra of Rt=5.316 min**

**MS/MS spectra of Rt=5.316 min**

**Rt=6.809 min**

**Primary MS spectra of Rt=6.809 min**

**7.** **chromatogram of possible dimeric products of neohesperidin dihydrochalcone when the formula [C_56_H_70_O_30_-H]^-^ was extracted.**

**
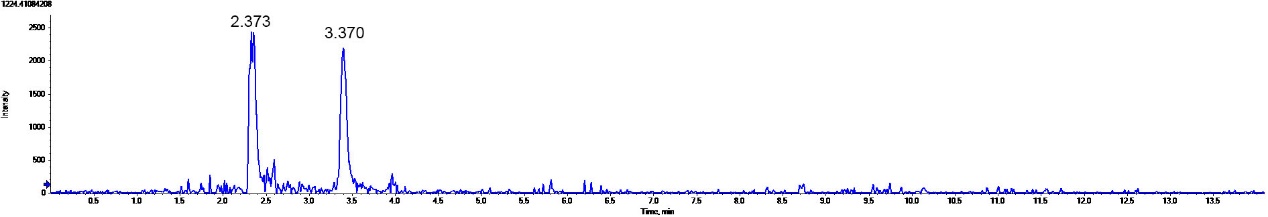
**

**Rt=2.373 min**

**Primary MS spectra of Rt=2.373 min**

**Rt=3.370 min**

**Primary MS spectra of Rt=3.370 min**

**
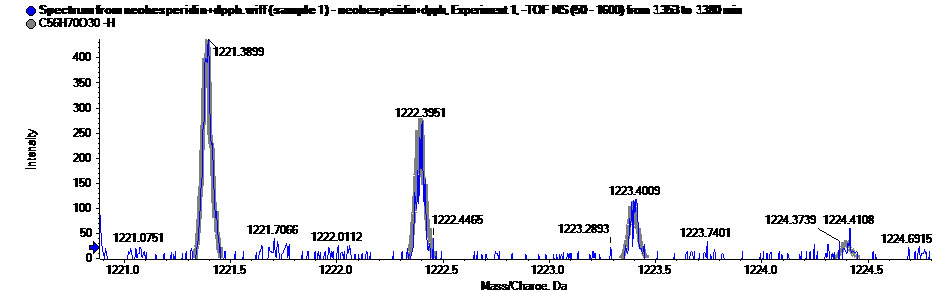
**
