# Supplementary material for: Antioxidant Structure–Activity Relationship Analysis of Five Dihydrochalcones
Source: Molecules. 2018 May 12;23(5):1162. doi: 10.3390/molecules23051162 (PMC6100071; doi:10.3390/molecules23051162)
Supplement: Supplementary file 1 [file molecules-23-01162-s001.zip › Supples/Suppl 3 Appearances and analysis certificates and of five dihydrochalcones.pdf]

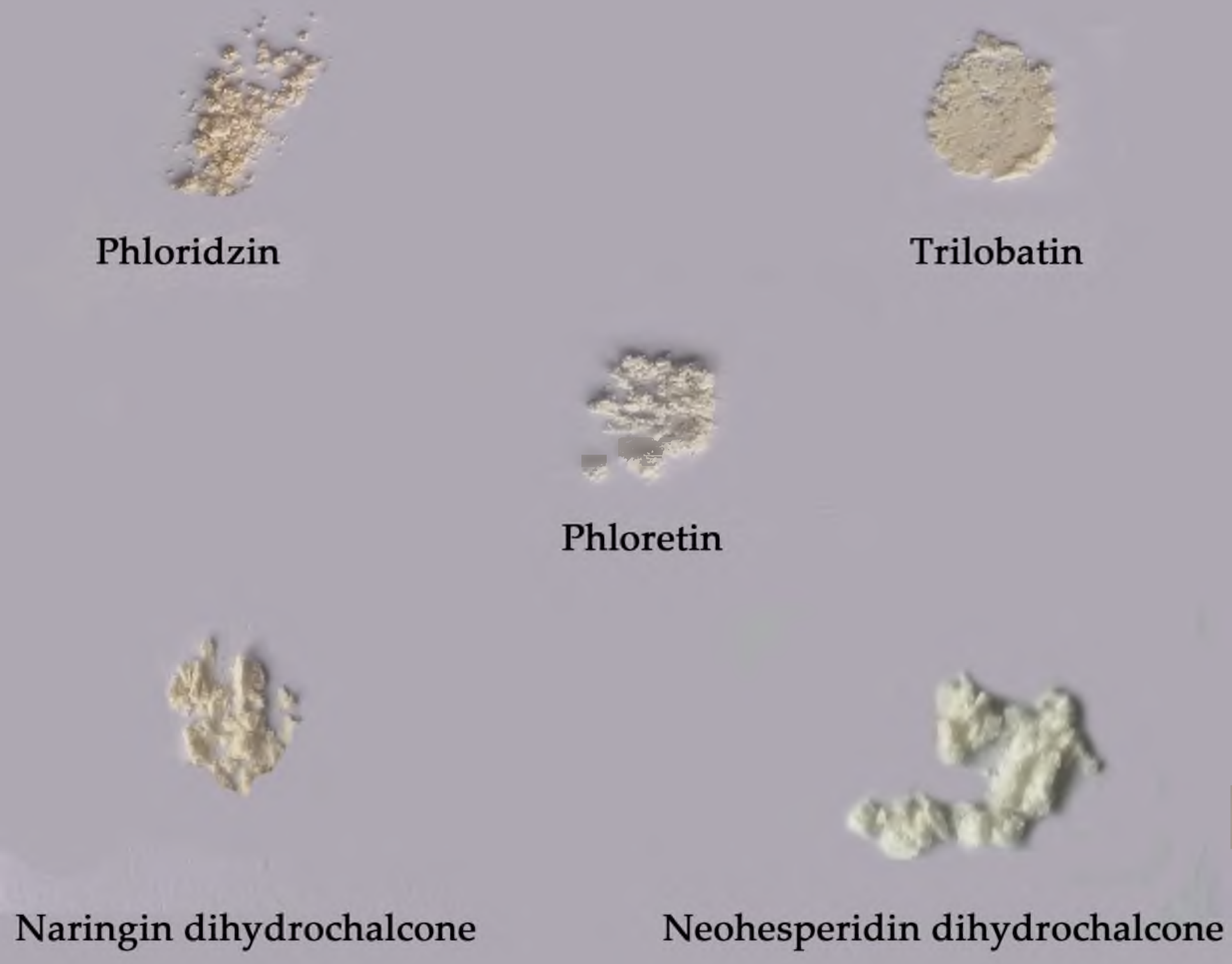
Phloridzin
Trilobatin
Phloretin
Naringin dihydrochalcone
Neohesperidin dihydrochalcone

产品分析证书
Certificate of Anaiysis

中文名称：根皮素

English Name：Phloretin
别名(Alias): Asebogenol; Dihydronaringenin; Phloretol

产品编码(Cat. No.):BP1088
CAS Number: 60-82-2
分子式(M. F.): $C_{15}H_{14}O_5$
分子量(M. W.): 274.272

批号(Batch No.): PRF9022842
报告日期(Report date): 2018-02-28

Structure

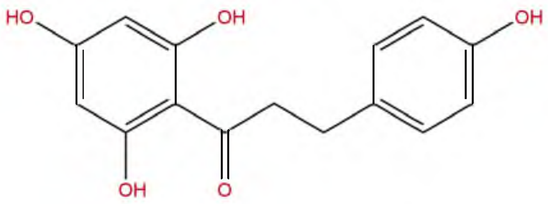

检验结果（Analytical result）:

**检验项目（Test Item）**	**检验指标（Specifications）**	**检验结果（Results）**
外观Appearance	Off-white powder	Off-white powder
干燥失重Lcss cn drylng	＜3.0%	1.23%
纯度Purity (HPLC-DAD,286nm)*	≥98.0%	99.29%
质谱Mass	274.2±1	Conforms
核磁NMR	Comply with the structure	Conforms

*** 色谱图见附件（Please find HPLC chromatography attached.）**

检测方法（Test Method）：Column: Agilent 5HC-C18，4.6*250mm，5.0um; **Column temperature**: 30℃; **Detectlon Mode:** UV286nm;**Flow Rate:** 1.0mi/min; **Sampie dissolutlon:** 30% Acetonitrile; **Mobiie Phase: A,** Acetonitrile **B,** 0.1% Phosphoric acid in water;**Gradient elution:**A, 30%-40%,15min.

贮存条件（Storage）:2~8℃, protected from light, keep package airproofed when not in use..

复测期（Retest date）:two years (2020-02-27) under conditions list above.

QC: Zhang Ling
Date: 2018-02-28

QA: Wu Qi
Date: 2018-02-28

备注(Remarks): The sample solutions should be prepared and used on the same day, it is the best preparing the solutions immediately before use. If the solutons have to be made up In advance, It should be made as allquots In tlghtly sealed vlals at less than -20℃. Generally, these mlght be useable for up to two weeks.

In case of quality issue, please contact us within 15 days after receipt of the product.

Empower 2 software

SAMPLE INFORMATION

Sampie Name:	Phloretin PRF9022842	Acqulred By:	System
Sampie Type:	Unknown	Sample Set Name:	
Vial:	102	Acq. Method Set:	Phloretin
injection #:	1	Processing Method:	sample
injection Volume:	10.00 ul	Channei Name:	286.0nm
Run Time:	25.0 Minutes	Proc. Chni. Descr.:	PDA 286.0 nm

Date Acquired: 2018-2-28 16:29:08 CST
Date Processed: 2018-2-28 17:08:06 CST

Auto-Scaled Chromatogram

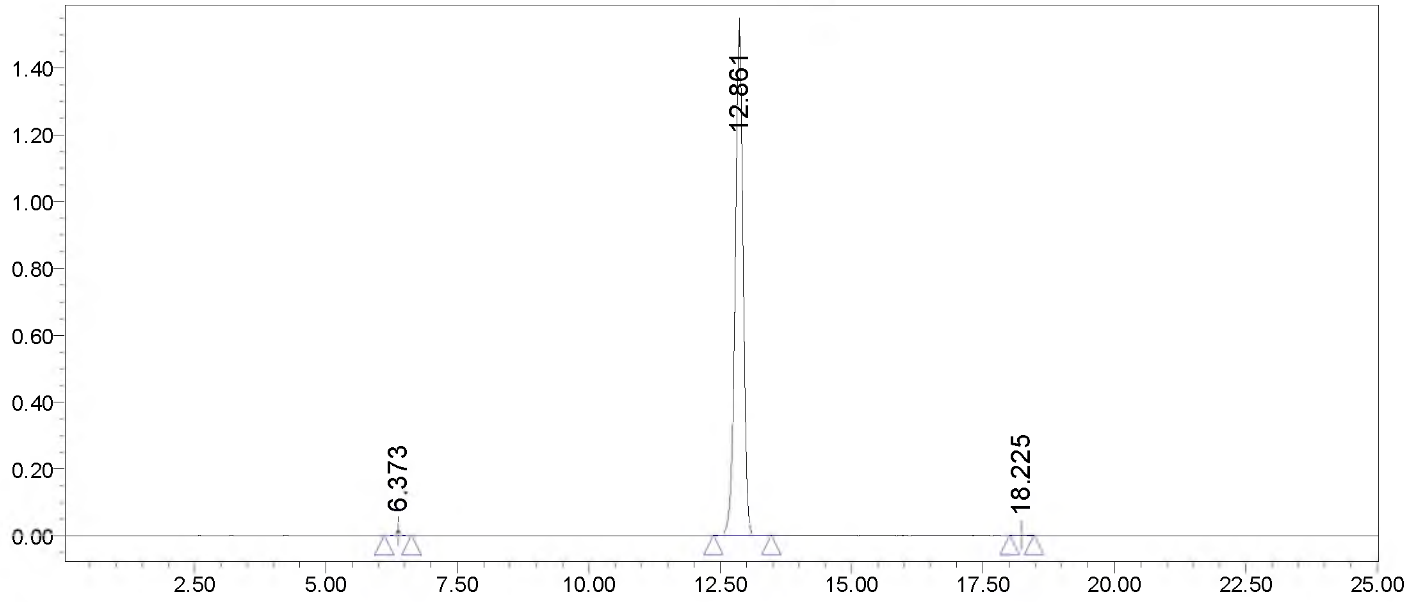

Peak Results

	RT	Area	% Area	USP Plate Count	USP Resolutlon
1	6.373	103574	0.64	17399.14	
2	12.861	16080134	99.29	34288.96	26.96
3	18.225	11534	0.07	63826.66	18.98

成都普瑞法科技开发有限公司
Chengdu Biopurify Phytochemicais Ltd.

Add: No.11 Building, No. 388 Rongtaidadao CNSTP
Wenjiang Zone, Chengdu, Sichuan, 611130 China
TEL: 028-82633987 FAX: 028-82633165
E-mail: biopurify@gmail.com sales@biopurify.com
Web: www.biopurify.com

Certificate of Analysis

Product Name: Phloridzin

Other Name:

Catalogne No.: BP1089

Batch No.: 15083102

Reported Date: 2015-08-31

CAS Nnmber: 60-81-1

Mol. Formnla: $C_{21}H_{24}O_{10}$

Mol. Weight: 436.413

Botanical Sonrce: Micromelum tephrocarpum

Type of componnd:

Identification Method: Mass, NMR

Analysis Method of Pnrity: HPLC-DAD

Structure:

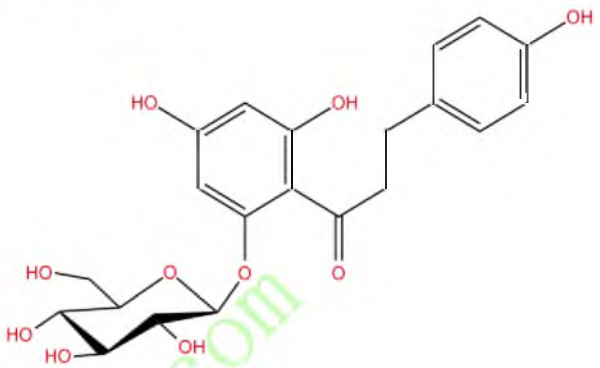

www.biopurify.com

Analytical resnlt:

Test	Specification	Resnlts
Appearance	Off-white powder	Off-white powder
Loss on drying	＜3.0%	1.1%
Purity (HPLC, 285nm)*	≥98.0%	99.20%

* Please find HPLC chromatography attached.

Package: Brown vial or HDPE Plastic Bottle

Storage: Cool and Dry place, protected from light, keep package airproofed when not in use.

Expiration: two years (2017-08-31) under conditions list above.

QC: MengPan **QA:** Lianglei Zhang

Date: 2015-08-31 **Date: 2015-08-31**

Empower2 software

Chengdu Biopurify Phytochemicais Ltd

SAMPLE INFORMATION

Sample Name:	Phloridzin 15083102	Acquired By:	panmeng
Sample Type:	Unknown	Sample Set Name:	
Vial:	1:C,2	Acq. Method Set:	Phloridzin
Injection #:	1	Processing Method:	Samples
Injection Volume:	10.00 ul	Channel Name:	285.0nm
Run Time:	20.0 Minutes	Proc. Chnl. Descr.:	PDA 285.0 nm
Date Acquired:	2015-8-31 9:37:48 CST		
Date Processed:	2015-8-31 10:37:03 CST		

Auto-Scaled Chromatogram

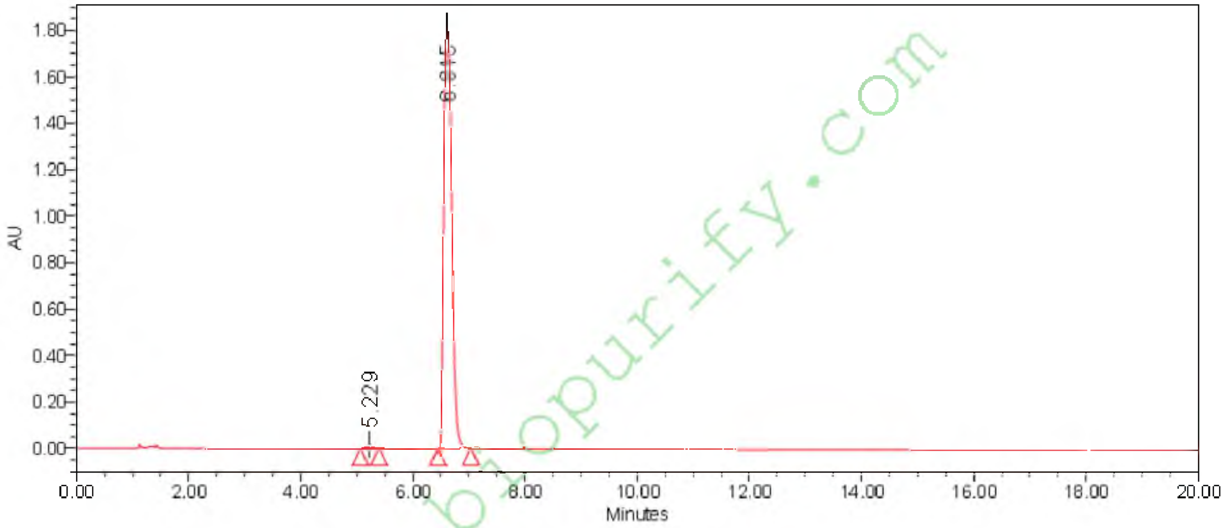

www.biopurify.com

Peak Results

	Name	RT	Area	% Area	USP Plate Count	USP Resolution
1		5.229	143606	0.80	9702.32	
2		6.615	17829822	99.20	10920.06	6.00

成都普瑞法科技开发有限公司
Chengdu Biopurify Phytochemicais Ltd.

Add: No.11 Building, No. 388 Rongtaidadao CNSTP
Wenjiang Zone, Chengdu, Sichuan, 611130 China
TEL: 028-82633987 FAX: 028-82633165
E-mail: biopurify@gmail.com sales@biopurify.com
Web: www.biopurify.com

Certificate of Analysis

Product Name: Trilobatin

Other Name:

Catalogne No.: BP1756

Batch No.: PRF15092821

Report date 2015-09-28

CAS Nnmber: 4192-90-9

Mol. Formnla: C21H24O10

Mol. Weight: 436.413

Type of compound:

Botanical Sonrce:

Identification Method: Mass, NMR

Analysis Method of Pnrity: HPLC-DAD

Structure:

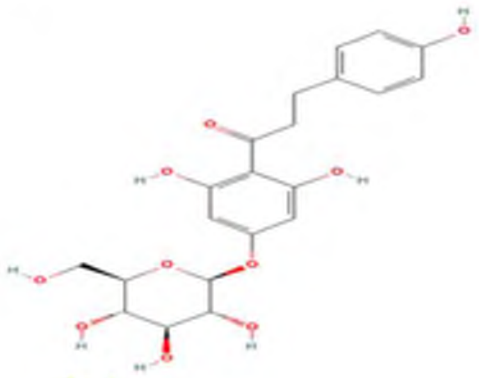

Analytical resnlt:

Test	Specification	Resnlts
Appearance	Pale Yellow powder	Pale Yellow powder
Loss on drying	<3.0%	1.26%
Purity (HPLC,282nm) *	≥98.0%	98.38%

* Please find HPLC chromatography attached.

Package: Brown vial or HDPE Plastic Bottle

Storage: Cool and Dry place, protected from light, keep package airproofed when not in use.

Expiration: two years (2017-09-28) under conditions list above.

QC: Meng Fan
Date: 2015-09-28

QA: Lianglei Zhang
Date: 2015-09-28

www.biopurify.c

Empower2 software

Chengdu Biopurify Phytochemicals Ltd.

SAMPLE INFORMATION

Sample Name:	Trilobatin PRF15092821	Acquired By:	System
Sample Type:	Unknown	Sample Set Name:	
Vial:	18	Acq. Method Set:	Trilobatin
Injection #:	1	Processing Method:	Samples
Injection Volume:	10.00 ul	Channel Name:	282.0nm
Run Time:	25.0 Minutes	Proc. Chnl. Descr.:	PDA 282.0 nm
Date Acquired:	2015-9-28 13:39:29 CST		
Date Processed:	2015-9-28 15:11:08 CST		

Auto-Scaled Chromatogram

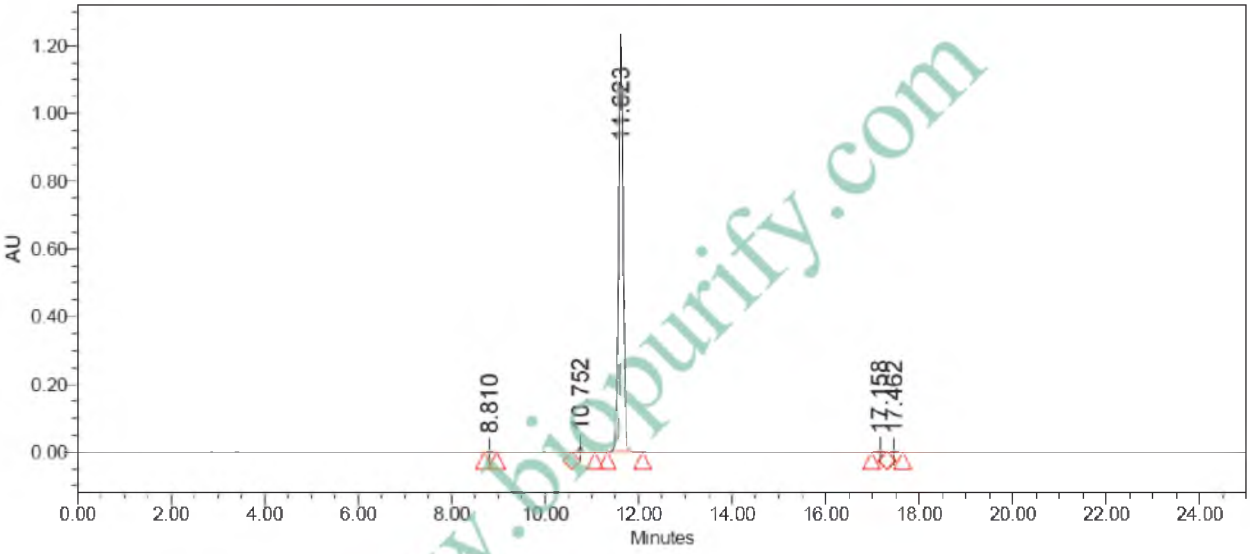

Peak Results

	Name	RT	Area	% Area	USP Plate Count	USP Resolution
1		8.810	[illegible]7610	0.21	39561.30	
2		10.752	80935	0.94	58864.50	10.78
3		11.623	8445589	98.38	63431.19	4.72
4		17.158	26384	0.31	106622.62	27.45
5		17.462	14429	0.17	149002.46	1.52

www.biopurify.com

成都普瑞法科技开发有限公司
Chengdu Biopurify Phytochemicals Ltd.

Add: No.11 Building, No. 388 Rongtaidadao CNSTP
Wenjiang Zone, Chengdu, Sichuan, 611130 China
TEL: 028-82633987 FAX: 028-82633165
E-mail: biopurify@gmail.com sales@biopurify.com
Web: www.biopurify.com

Certlficate of Analysis

Product Name: Neohesperidin Dihydrochal

Other Name: Neohesperidine Dihydrochalcone

Catalogue No.: BP0992

Batch No.: PRF7081416

Report date: 2016-08-14

CAS Number: 20702-77-6

Mol. Formula: $C_{28}H_{36}O_{15}$

Mol. Weight: 612.581

Type of compound:

Identification Method: Mass, NMR

Analysis Method of Purity: HPLC-ELSD

Structure:

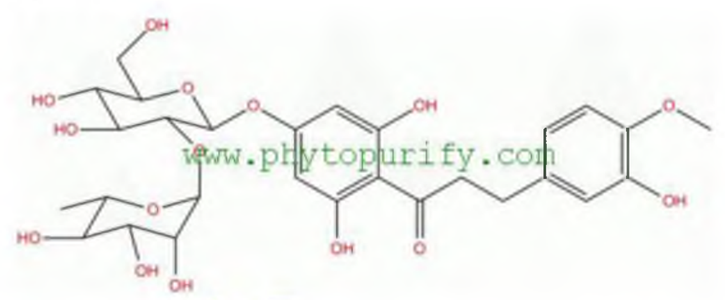

www.biopurify.com

Analytical result:

**Test**	**Specification**	**Results**
Appearance	Off-white Powder	Off-white Powder
Loss on drying	＜3.0%	1.2%
Purity (HPLC, ELSD)*	≥98.0%	99.39 %

* Please find HPLC chromatography attached.

Package: Brown vial or HDPE Plastic Bottle

Storage: Cool and Dry place, protected from light, keep package airproofed when not in use.

Expiration: two years (2018-08-14) under conditions list above.

QC: Meng Fan **QA:** Lianglei Zhang

Date: 2016-08-14 **Date:** 2016-08-14

Data File D:\HPLC\DATA\NEOHESPERIDIN DIHYDROCHALCONE\PRF7081416.D
Sample Name: Neohesperidin Dihydrochalcone

===

Acq. Operator : yzq
Acq. Instrument : Instrument 1 Location : Vial 2
Injection Date : 8/14/2016 1:00:51 PM
Inj Volume : 10 µl
Acq. Method : D:\YJH\METHOD\NEOHESPERIDIN DIHYDROCHALCONE.M
Last changed : 8/14/2016 12:55:47 PM by yzq
Analysis Method : D:\YJH\METHOD\HYPEROSIDE .M
Last changed : 8/14/2016 1:25:21 PM by yzq
(modified after loading)

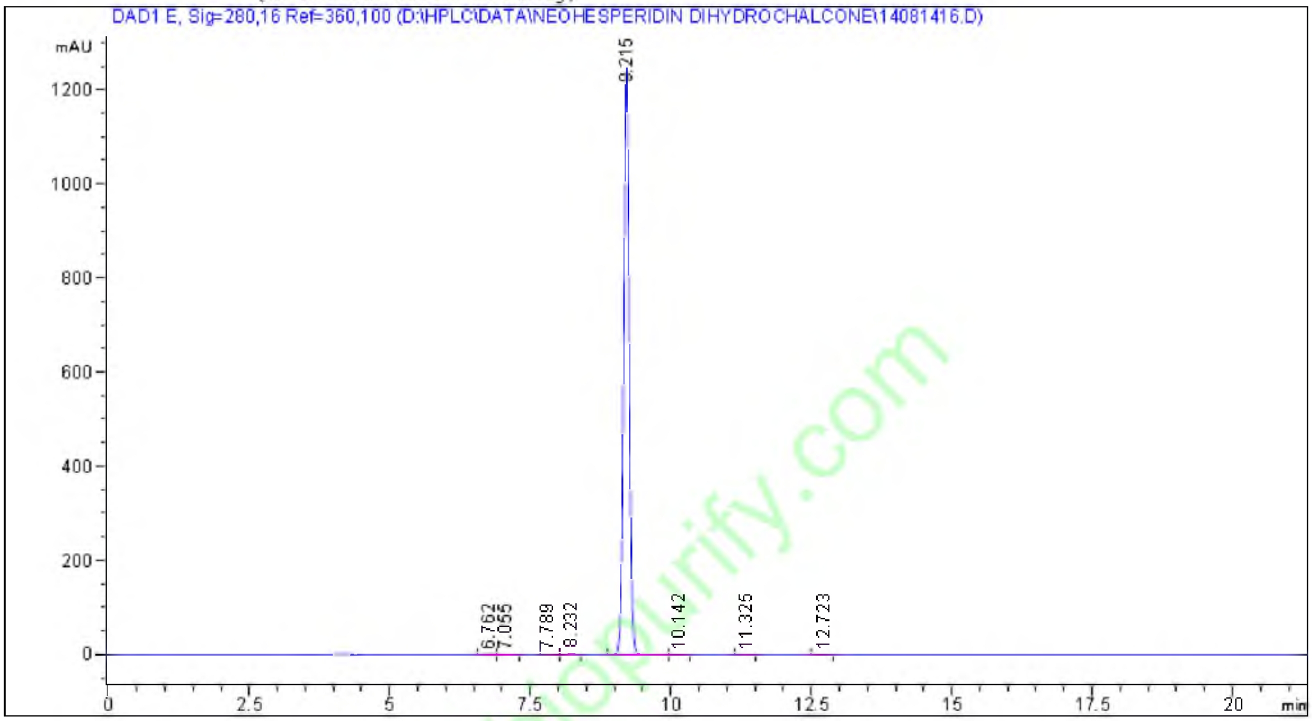

www.biopurify.com

===

Area Percent Report

===

Sorted By : Signal
Multiplier : 1.0000
Dilution : 1.0000
Use Multiplier & Dilution Factor with ISTDs

Signal 1: DAD1 E, Sig=280,16 Ref=360,100

Peak #	RetTime [min]	Type	Width [min]	Area [mAU*s]	Height [mAU]	Area %
1	6.762	VV	0.1148	3.16273	4.15340e-1	0.0365
2	7.055	VV	0.1416	5.16198	5.03571e-1	0.0596
3	7.789	VV	0.1310	2.69115	2.99090e-1	0.0311
4	8.232	VV	0.1035	26.01729	3.81641	0.3003
5	9.215	VV	0.1065	8611.60742	1248.48096	99.3979
6	10.142	VV	0.1489	5.99620	5.60277e-1	0.0692
7	11.325	VV	0.1163	5.72776	7.23783e-1	0.0661
8	12.723	VV	0.1753	3.40924	2.70096e-1	0.0394

Totals : 8663.77376 1255.06952

产品分析证书
Certificate of Analysis

中文名称：柚皮苷二氢查尔酮

English Name：Naringin dihydrochalcone
别名**(Allas):** Naringin DC

产品编码**(Cat. No.):**BP0984
CAS Number: 18916-17-1
分子式**(M. F.):** C27H34O14
分子量**(M. W.)**: 582.555

批号**(Batch No.):** PRF7083123
报告日期**(Report date): 2016/8/31**

Structure

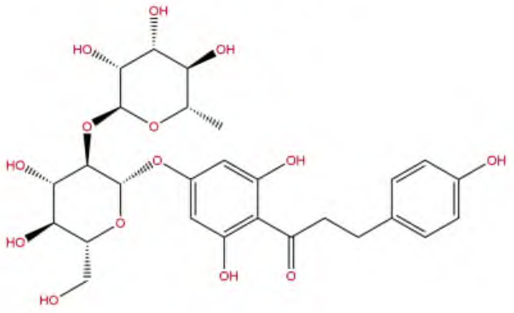

检验结果（**Analytical result**）：

检验项目（**Test Item**）	检验指标（**Specifications**）	检验结果（**Results**）
外观Appearance	Off-white powder	Off-white powder
干燥失重Loss on drying	＜3.0%	1.24%
纯度Purity (HPLC-DAD, 280nm)*	≥98.0%	99.58%
质谱Mass	582.555±1	Conforms
核磁NMR	Comply with the structure	Conforms

* 色谱图见附件（**Please find HPLC chromatography attached.**）

贮存条件（Storage）:2~8℃

复测期（Retest date）:two years (2018-08-30) under conditions list above.

备注（Remarks）：如遇质量问题，请于收到产品之日起 15 日内与我们联系。

In case of quality issue, please contact us within 15 days after receipt of the product.

QC: Zhang Ling　　　　QA: Wu Qi

Date: 2016年8月31日　　　　Date: 2016年8月31日

Tel：+86-28-82633987　Fax: +86-28-82633165
http://www.biopurify.com　Email: sales@biopurify.com　biopurify@gmail.com

Chengdu Biopurify Phytochemicals Ltd.

SAMPLE INFORMATION

Sampie Name:	Naringin dihydrochaicone	Acquired By:	System
Sampie Type:	Unknown	Sampie Set Name:	
Vial:	47	Acq. Method Set:	Naringin dihydrochaicone
Injection #:	1	Processing Method:	Samples
Injection Voiume:	5.00 ui	Channei Name:	280.0nm
Run Time:	25.0 Minutes	Proc. Chni. Descr.:	PDA 280.0 nm
Date Acquired:	2016-8-31 11:22:53 CST		
Date Processed:	2016-8-31 12:44:08 CST		

Auto-Scaled Chromatogram

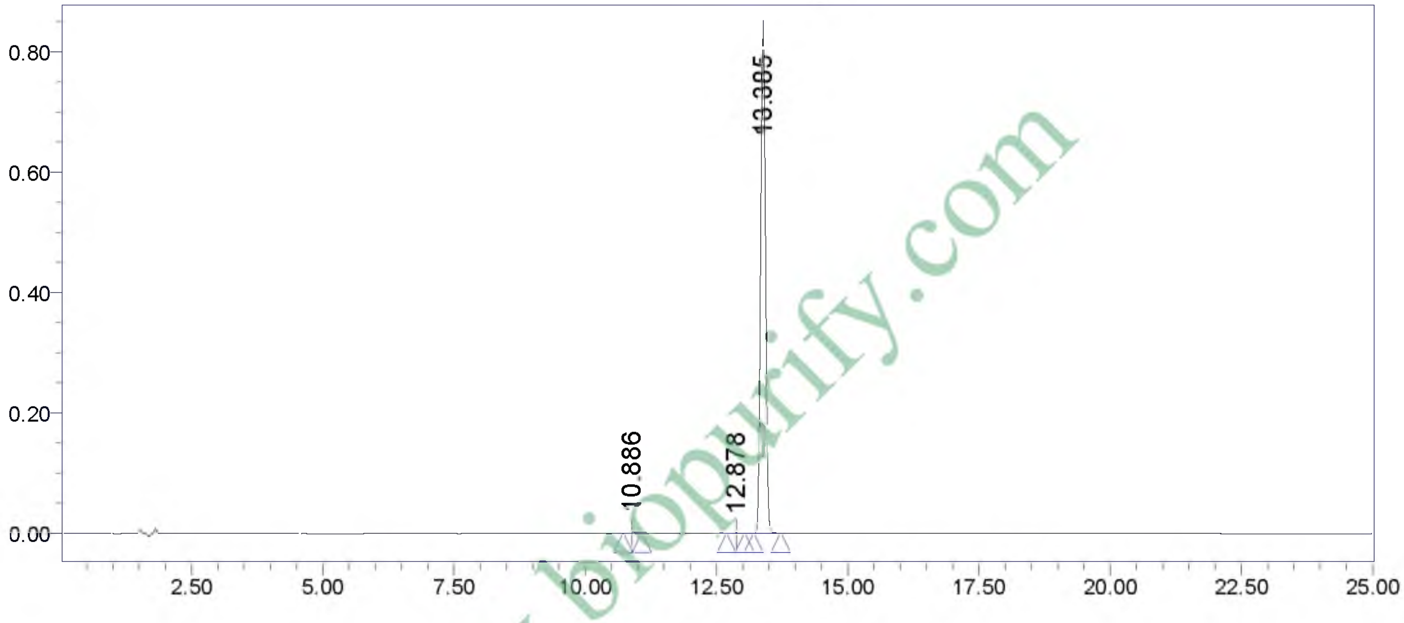

www.biopurify.com

Peak Results

	RT	Area	% Area	USP Plate Count	USP Resolution
1	10.886	18809	0.35	39098.77	
2	12.878	4012	0.07	70154.13	9.38
3	13.385	5400028	99.58	94752.10	2.68
