# Supplementary material for: Antioxidant Structure–Activity Relationship Analysis of Five Dihydrochalcones
Source: Molecules. 2018 May 12;23(5):1162. doi: 10.3390/molecules23051162 (PMC6100071; doi:10.3390/molecules23051162)
Supplement: Supplementary file 1 [file molecules-23-01162-s001.zip › Supples/Suppl 1 Dose response curves.doc]

**Supplementary Materials-1 Dose response curves**

**Antioxidant Structure-Activity Relationship Analysis of Five Dihydrochalcones**

**Xican Li ^1, 2, *^, Ban Chen ^1, 2^, Hong Xie ^1, 2^, Yuhua He ^1^**, **Dewei Zhong ^1^, and Dongfeng Chen ^3, 4, *^**

^1^	School of Chinese Herbal Medicine, Guangzhou University of Chinese Medicine, Waihuan East Road No. 232, Guangzhou Higher Education Mega Center, Guangzhou 510006, China. [505780526@qq.com](mailto:505780526@qq.com) (Y.H.); [393916211@qq.com](mailto:393916211@qq.com) (D.Z.); [xiehongxh1@163.com](mailto:xiehongxh1@163.com) (H.X.); [imchenban@foxmail.com](mailto:imchenban@foxmail.com) (B.C.)

^2^	Innovative Research & Development Laboratory of TCM, Guangzhou University of Chinese Medicine Waihuan East Road No. 232, Guangzhou Higher Education Mega Center, Guangzhou 510006, China.

^3^	School of Basic Medical Science, Guangzhou University of Chinese Medicine, Waihuan East Road No. 232, Guangzhou Higher Education Mega Center, Guangzhou 510006, China.

^4^	The Research Center of Basic Integrative Medicine, Guangzhou University of Chinese Medicine, Waihuan East Road No. 232, Guangzhou Higher Education Mega Center, Guangzhou 510006, China.

^*^ Corresponding author. E-mail: [lixican@126. com](mailto:lixican@126.com)(X.L.); lixc@gzucm.edu.cn(X.L.); chen888@gzucm.edu.cn; Tel.: +86-203-935-8076 (X.L.)

**Note:** This Supporting Information provides the original data of Tab. 1 in the main text. All data underline are mentioned in **Tab. 1** in the main text.

NAD, naringin dihydrochalcone; NED, neohesperidin dihydrochalcone.

1. FRAP Assay

Fig. S1.1 The does response curves of five dihydrochalcones and the positive control in FRAP assay.

Tab. S1.1 The comparison of IC_50_ values of five dihydrochalcones and positive control in FRAP assay, μg/mL (μM)

|  | 1 | 2 | 3 | **Mean±SD** |
| --- | --- | --- | --- | --- |
| Phloretin | 165.6  (603.9) | 211.2  (770.1) | 171.5  (625.3) | 182.8±24.8^b^  (666.4±90.4^B^) |
| Phloridzin | 1457.2  (3339.0) | 1293.3  (2963.4) | 1120.3  (2567.2) | 1290.3±168.4^d^  (2956.5±385.9^D^) |
| Trilobatin | 1052.1  (2410.7) | 916.9  (2100.9) | 1010.0  (2314.3) | 993.0±69.2^c^  (2275.3±158.5^C^) |
| NED | 52.7  (86.0) | 54.1  (88.3) | 65.4  (106.8) | 57.4±7.0^a^  (93.7±11.4^A^) |
| NAD | 1518.8  (2607.1) | 1644.7  (2823.2) | 1569.6  (2694.4) | 1544.2±35.9^e^  (2650.7±61.7^D^) |
| Trolox | 38.5  (153.6) | 30.8  (123.0) | 33.3  (133.3) | 34.2±3.9^a^  (136.6±15.6^A^) |

IC_50_ value was defined as the concentration of 50% superoxide anion radical inhibition and calculated by linear regression which was analyzed by Origin 6.0 professional software. Means values with different superscripts in the same column are significantly different (*p<*0.05).

2. ABTS^+^· Scavenging Assay

Fig. S1.2 The does response curves of five dihydrochalcones and the positive control in ABTS^+^· scavenging assay.

Tab. S1.2 The comparison of IC_50_ values of five dihydrochalcones and positive control in ABTS^+^· scavenging assay, μg/mL(μM)

|  | 1 | 2 | 3 | **Mean±SD** |
| --- | --- | --- | --- | --- |
| Phloretin | 0.6  (2.3) | 1.4  (5.1) | 1.5  (5.5) | 1.2±0.5^a^  (4.3±1.8^A^) |
| Phloridzin | 29.3  (67.0) | 27.8  (63.5) | 29.3  (67.0) | 28.7±0.9^d^  (65.8±2.0^E^) |
| Trilobatin | 7.9  (18.8) | 7.5  (17.1) | 7.4  (16.9) | 7.6±0.3^b^  (17.4±0.7^B^) |
| NED | 12.7  (20.8) | 12.5  (20.4) | 12.5  (20.4) | 12.6±0.2^c^  (20.5±0.2^B^) |
| NAD | 14.5  (24.8) | 15.1  (25.9) | 12.4  (21.3) | 14.0±1.4^c^  (24.0±2.4^C^) |
| Trolox | 7.5  (30.1) | 9.2  (36.8) | 8.0  (32.0) | 8.3±0.9^b^  (33.0±3.54^D^) |

IC_50_ value was defined as the concentration of 50% superoxide anion radical inhibition and calculated by linear regression which was analyzed by Origin 6.0 professional software. Means values with different superscripts in the same column are significantly different (*p<*0.05). Means values with different superscripts in the same column are significantly different (*p<*0.05).

3. DPPH· Scavenging Assay

Fig. S1.3 The does response curves of five dihydrochalcones and the positive control in DPPH· scavenging assay

Tab. S1.3 The comparison of IC_50_ values of five dihydrochalcones and positive control in DPPH· scavenging assay, μg/mL(μM)

|  | 1 | 2 | 3 | **Mean±SD** |
| --- | --- | --- | --- | --- |
| Phloretin | 18.1  (66.1) | 15.9  (58.3) | 18.1  (66.1) | 17.4±1.3^b^  (63.5±4.6^B^) |
| Phloridzin | 261.8  (599.9) | 234.2  (536.7) | 238.5  (546.6) | 244.8±14.8^e^  (561.0±34.0^E^) |
| Trilobatin | 71.5  (163.9) | 72.2  (165.5) | 71.0  (162.6) | 71.6±0.6^c^  (164.0±1.4^C^) |
| NED | 30.6  (50.0) | 40.3  (65.7) | 36.2  (59.2) | 35.7±4.8^b^  (58.3±7.9^B^) |
| NAD | 199.0  (341.6) | 179.2  (307.6) | 179.2  (307.7) | 185.8±11.4^d^  (318.9±19.6^D^) |
| Trolox | 3.5  (13.8) | 3.2  (13.0) | 3.3  (13.4) | 3.4±0.1^a^  (13.4±0.4^A^) |

IC_50_ value was defined as the concentration of 50% superoxide anion radical inhibition and calculated by linear regression which was analyzed by Origin 6.0 professional software. Means values with different superscripts in the same column are significantly different (*p<*0.05).

4. ·O_2_^-^ scavenging Assay

Fig. S1.4 The does response curves of five dihydrochalcones and the positive control in ·O_2_^-^ scavenging assay

Tab. S1.4 The comparison of IC_50_ values of five dihydrochalcones and positive control in ·O^2-^ scavenging assay, μg/mL(μM)

|  | 1 | 2 | 3 | **Mean±SD** |
| --- | --- | --- | --- | --- |
| Phloretin | 28.2  (102.7) | 29.4  (107.1) | 31.6  (115.3) | 29.7±1.8^a^  (108.4±6.4^A^) |
| Phloridzin | 144.9  (332.0) | 185.2  (424.3) | 174.3  (399.3) | 168.1±20.8^c^  (385.2±47.8^C^) |
| Trilobatin | 128.8  (295.2) | 118.4  (271.3) | 107.0  (245.1) | 118.1±10.9^b^  (270.6±25.0^B^) |
| NED | 33.4  (54.5) | 35.5  (57.9) | 34.0  (55.4) | 34.3±1.1^a^  (56.0±1.7^A^) |
| NAD | 185.8  (319.0) | 200.6  (344.1) | 177.8  (305.2) | 188.1±11.6^c^  (322.8±19.9^C^) |
| Trolox | 231.6  (925.2) | 229.2  (915.8) | 255.1  (1019.1) | 238.6±14.3^d^  (953.4±57.1^D^) |

IC_50_ value was defined as the concentration of 50% superoxide anion radical inhibition and calculated by linear regression which was analyzed by Origin 6.0 professional software. Means values with different superscripts in the same column are significantly different (*p<*0.05).
